# Fixed and Removable Bridgework

**Published:** 1922-04

**Authors:** 


### FIXED AND REMOVABLE BRIDGEWORK

There is a place for both forms of bridgework, just as there is a place and demand for the gold foil filling and the gold inlay. There are also many different methods of special practice, such as plate dentures, root-canal treatments, pyorrhea treatments, etc. We long since discovered that some dentists could get more satisfactory results with other methods than any set or standardized procedure. This is well, as it makes for a wider field of culture and versitility, and it prevents pedantry and dogmatism. If we can keep our technical methods abreast of the times and faithfully study our problems scientifically, and resist the temptations to commercialism, there is no reason why we should fear any intimation that our profession is not to become a real force in the promotion of human health and welfare.

